# Efficacy of sorafenib in patients with gastrointestinal stromal tumors in the third- or fourth-line treatment: A retrospective multicenter experience

**DOI:** 10.3892/ol.2013.1408

**Published:** 2013-06-17

**Authors:** UMUT KEFELI, MUSTAFA BENEKLI, ALPER SEVINC, RAMAZAN YILDIZ, MUHAMMED ALI KAPLAN, AYDIN CILTAS, OZAN BALAKAN, ABDURRAHMAN ISIKDOGAN, UGUR COSKUN, FAYSAL DANE, HAKAN HARPUTLUOGLU, HALIT KARACA, DOGAN YAZILITAS, AYSE DURNALI, ALI OSMAN KAYA, UMUT DEMIRCI, MAHMUT GUMUS, SULEYMAN BUYUKBERBER

**Affiliations:** 1 Department of Medical Oncology, Medeniyet University Goztepe Training and Research Hospital, Istanbul;; 2 Department of Medical Oncology, Gazi University Faculty of Medicine, Ankara;; 3 Department of Medical Oncology, Gaziantep University Faculty of Medicine, Gaziantep;; 4 Department of Medical Oncology, Dr. Lutfi Kirdar Kartal Education and Research Hospital, Istanbul;; 5 Department of Medical Oncology, Dicle University Faculty of Medicine, Diyarbakir;; 6 Department of Medical Oncology, Marmara University Faculty of Medicine, Istanbul;; 7 Department of Medical Oncology, Inonu University Faculty of Medicine, Malatya;; 8 Department of Medical Oncology, Erciyes University Faculty of Medicine, Kayseri;; 9 Department of Medical Oncology, Konya Education and Research Hospital, Konya;; 10 Department of Medical Oncology, Dr. A.Y. Ankara Oncology Education and Research Hospital, Ankara;; 11 Department of Medical Oncology, Medicana Bahcelievler Hospital, Istanbul;; 12 Department of Medical Oncology, Ataturk Education and Research Hospital, Ankara, Turkey

**Keywords:** sorafenib, gastrointestinal stromal tumors, efficacy

## Abstract

Sorafenib is a multi-targeted tyrosine kinase receptor inhibitor used to treat patients with advanced gastrointestinal stromal tumors (GISTs). The present study evaluated the efficacy and tolerability of sorafenib therapy for patients with GISTs. Between January 2001 and November 2012, 25 patients, from multiple centers, who had received sorafenib as the third- or fourth-line treatment for GISTs were investigated retrospectively. In total, 17 patients were male and eight were female. The median age was 54.0 years (range, 16–82 years). From the patients, 21 received imatinib for longer than six months and four received it for less than six months. The clinical benefit rate of sorafenib was 40.0%. Treatment-related adverse events were reported in 72% of patients. These adverse events were generally mild to moderate in intensity. The median progression-free survival (PFS) and overall survival (OS) times of the patients who received sorafenib were 7.2 and 15.2 months, respectively. The duration of imatinib usage was an independent prognostic factor for PFS and OS. Sorafenib is an effective treatment in patients with GISTs showing a clinical benefit rate of 40.0% and an acceptable tolerability.

## Introduction

Gastrointestinal stromal tumors (GISTs) are the most common mesenchymal neoplasms of the gastrointestinal tract and are highly resistant to conventional chemotherapy (1). C-kit (also known as CD117) expression occurs in ∼95% of GISTs, thereby enabling differentiation from other mesenchymal spindle-cell neoplasms (2). C-kit is a transmembrane receptor that is activated by binding of the KIT ligand, a stem cell factor. Of all the GISTs, ∼85–90% are associated with gain-of-function KIT gene mutations that lead to the constitutive activation of KIT kinase activity. A significantly smaller proportion (5%) are associated with analogous gain-of-function mutations in PDGFRA, the gene encoding platelet derived growth factor receptor α (PDGFRα); <10% contain no identified receptor tyrosine kinase mutations (3–5). Experience gained from epidemiological studies and active GIST therapeutic trials suggests that the annual incidence of GISTs in the United States is at least 4,000 to 6,000 new cases (roughly seven to 20 cases per million population) per year (6).

Surgical resection remains the mainstay therapy for GISTs, but recurrence is common. The five-year survival rates for GISTs following complete resection range between 40 and 65% (7–11). Imatinib mesylate selectively inhibits certain protein tyrosine kinases; intracellular ABL kinase, chimeric BCR-ABL fusion oncoprotein of chronic myeloid leukemia, the transmembrane receptor KIT and platelet-derived growth factor receptors (PDG-FR) (12–15). Imatinib mesylate has induced a sustained objective response in >50% of patients with advanced GISTs in Western and other countries (16–18). However, the response to imatinib therapy is time-limited and secondary resistance to imatinib therapy (following initial stabilization or response) develops in the majority of patients (19).

Sunitinib malate is an oral multi-targeted receptor tyrosine kinase inhibitor that has shown antiangiogenic and antitumor activities in several *in vitro* and *in vivo* tumor models (7,20–25). Sunitinib has shown effective activity for patients with GISTs after imatinib failure or intolerance, and has induced a sustained clinical benefit in advanced GISTs (7,18). A number of imatinib-resistant mutations confer cross-resistance to sunitinib. Therefore, various agents, including sorafenib, have been tested as salvage therapy for patients with these resistant GISTs (26). In a prospective multicenter phase II study involving patients with unresectable, KIT-positive GISTs that had progressed under imatinib and sunitinib treatment, 55% of patients who received sorafenib had stable disease and 13% had partial responses (27). In a retrospective analysis of 32 patients, sorafenib was shown to be significantly active in patients with metastatic GISTs resistant to imatinib and sunitinib (28).

Based on the limited data, guidelines have included sorafenib as an option for patients who are no longer receiving a clinical benefit from imatinib or sunitinib (29). Therefore, the aim of the present study was to report the results of sorafenib treatment in Turkish GIST patients.

## Materials and methods

### Patients and study design

A total of 250 patients with GISTs from ten institutions in Turkey were retrospectively evaluated. All cases of surgically or endoscopically resected GISTs, investigated by the pathology departments of the participating institutions (between January 2001 and November 2012), were reviewed. Of these, the cases of 25 patients who received sorafenib as the third- or fourth-line treatment from eight institutions were selected for evaluation according to the Response Evaluation Criteria in Solid Tumors (RECIST) (30). Follow-up data were obtained from clinical records and histopathology reports. Written informed consent was obtained from all patients. The GISTs were defined as primary spindle cell, epithelioid cell and mixed neoplasms of the tubular GI tract with an overexpression of CD117 and with or without CD34 expression, according to well-established criteria for GIST diagnosis (31,32). Mitoses were counted in 50 high-power fields. Tumor sizes were recorded as the largest diameter in any dimension of the primary tumor and were classified as <2, 2–5, 5–10 or >10 cm. The malignant potential of the GISTs was classified according to the risk categories proposed by Fletcher *et al* (32). The patient, tumor and treatment variables were recorded. Patient data included age, gender and presentation status. All patients with any metastatic disease were considered to have a metastatic presentation, regardless of whether they had received prior therapy or had also had local recurrence. All tumors were regarded as being histologically malignant.

### Statistical analysis

All data were analyzed using SPSS 17.0 software (SPSS Inc., Chicago, IL, USA). Based on the low number of patients, non-parametric tests were selected for the evaluation. Actuarial survival was determined by Kaplan-Meier analysis. Tumor response rates were evaluated as complete response (CR), partial response (PR), stable disease (SD) and progressive disease (PD) according to the RECIST criteria (30). CR, PR and SD were accepted as a response to sorafenib treatment, while PD was accepted as a non-responsive to sorafenib treatment. The duration of imatinib usage was recorded as more or less than six months. Progression free survival (PFS) was defined as no progression after sorafenib use. Overall survival (OS) was defined as survival following the administration of sorafenib and mortality was the endpoint of the study. The associations of patient, tumor and treatment characteristics with outcome were tested by univariate analysis using a log-rank test. A multivariate analysis was performed using the Cox proportional hazards model, and only variables that were deemed statistically significant were included in the final Cox model. Multivariate P-values were used to characterize the independence of these factors. The 95% confidence interval (CI) was used to quantify the association between survival time and each independent factor. All P-values were two-sided in the tests and P<0.05 was considered to indicate a statistically significant difference.

## Results

### Clinical features

Between January 2001 and November 2012, a total of 250 patients with GISTs were evaluated and 25 who had treatment failure with imatinib and other thyrosine-kinases inhibitors were included in the present study. All patients were previously treated with imatinib. Of these, 17 (68.0%) were male and eight (32.0%) were female. The median age was 54.0 years (range, 16–82 years) and the peak age was between 40 and 60.

Of the GIST patients, 77% were diagnosed as clinically symptomatic. The most commonly presented symptoms were abdominal pain and non-specific symptoms due to an abdominal mass (55.5%). The tumors most commonly originated in the stomach (20.0%) and the small intestine (64.0%). Histologically, the majority of tumors were predominantly spindle-shaped (36.0%) or of mixed type (36.0%). All of the tumors were CD117-positive, while 68.0% were CD34-positive. A total of 17 patients (68.0%) presented with metastasis at diagnosis. Among these patients, 11 of the metastasis sites were the liver (64.7%), three were the peritoneum (17.6%), one was the lung (5.9%) and two were other sites (11.8%). In total, 21 patients (84.0%) received imatinib for longer than six months, while four (16.0%) received it for less than six months (Table I).

The majority of the tumors (72.0%) were >10 cm in size. Based on the size of the primary tumor, the localization and the mitotic index, 24.0% of the patients were classified as intermediate-risk and 76.0% as high-risk according to the NIH risk classification. There were no extremely low- or low-risk groups (Table II).

### Treatment outcomes

Sorafenib was administered to 25 patients and all patients were followed up after the administration at regular intervals until mortality or the time this manuscript was written. The dosage of sorafenib was 2× 400 mg/day at the beginning of the treatment. Treatment was continued until the patient no longer clinically benefitted from therapy or until unacceptable toxicity occurred. Temporary dose interruption and/or dose reduction of sorafenib therapy was provided if an intolerance or any adverse effects occurred.

A total of 18 (72.0%) patients received 400 mg/day imatinib and seven patients (28.0%) with PD received 600–800 mg/day imatinib in second-line treatment. All the patients had PD during imatinib treatment and no patients were intolerant to imatinib.

Of the patients, 12 received sorafenib in the third-line and 13 received it in the fourth-line treatment. Nine of the patients who received sorafenib in the fourth-line received sunitinib and three received nilotinib in the third-line treatment. Overall, 10 (40%) of the patients achieved responses while receiving sorafenib. This represents the clinical benefit of sorafenib as determined by the sum of PR and SD. No patients achieved CR. Six patients (24.0%) achieved PR and four (16.0%) achieved SD during sorafenib usage. A total of 15 (60%) patients showed PD at the time of analysis according to RECIST.

**Figure 1.**
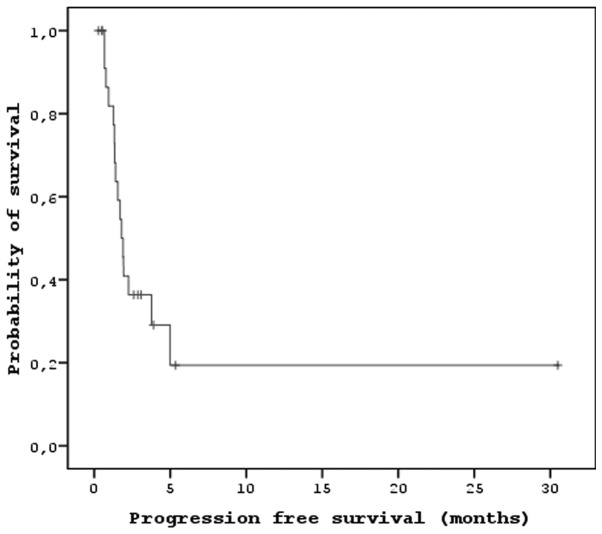
Progression-free survival (PFS) of the 25 patients that received sorafenib.

**Figure 2.**
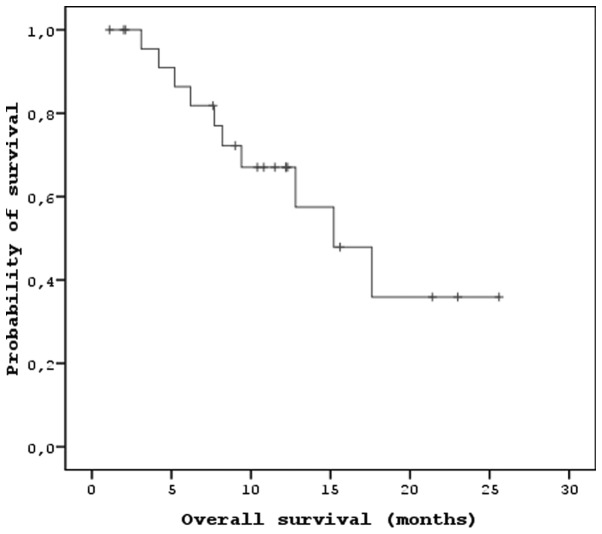
Overall survival (OS) of the 25 patients that received sorafenib.

**Figure 3.**
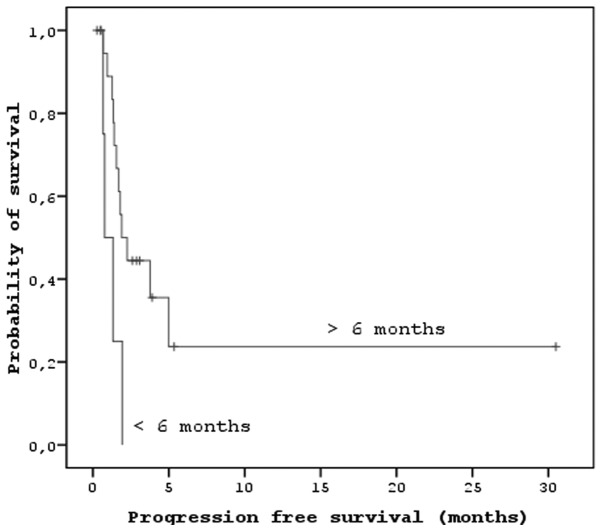
Univariate analysis between the duration of imatinib usage and progression-free survival (PFS). Patients received imatinib for either more or less than six months.

**Figure 4.**
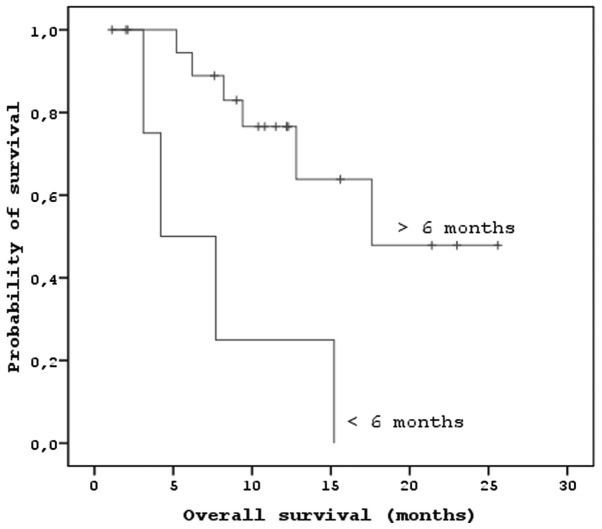
Univariate analysis between the duration of imatinib usage and overall survival. Patients received imatinib for either more or less than six months.

The median PFS and OS times of the patients that received sorafenib were 7.2 months (95% CI, 5.47–8.92) and 15.2 months (95% CI, 9.26–21.13), respectively (Figs. 1 and 2). In the univariate analysis, there were significant correlations between localization (P<0.01) and PFS. There were also significant correlations between the duration of imatinib usage, the response to sorafenib and the PFS and OS (P<0.05; Figs. 3 and 4). There were no associations between age, gender, tumor risk category, imatinib dose, duration of sunitinib usage and PFS and OS (P>0.05). In the multivariate analysis, there were significant associations between the duration of imatinib usage and PFS (P<0.05) and OS (P<0.05; Tables III and IV).

Treatment-related adverse events were reported in 72% of the patients. These adverse events were generally mild to moderate in intensity and managed by dose reduction or standard supportive medical treatments. Hypertension only occurred in one patient and this was managed with anti-hypertensive drugs. None of the patients discontinued sorafenib treatment due to adverse events. The most common adverse events of any grade were skin rashes (54%), thrombocytopenia (34%) and fatigue (38%). The most common grade III side-effect was hand-foot syndrome (HFS; 38%); 41% of these patients received dose reductions due to HFS.

## Discussion

Approximately 50% of patients with GIST eventually develop progression within 24 months of imatinib treatment (33). Patients with advanced GIST who undergo disease progression or are intolerant to first-line imatinib therapy usually start second-line sunitinib malate therapy. As has been observed for imatinib in a first-line setting, the majority of patients showing an initial clinical benefit from sunitinib develop PD (34). In a prospective multicenter phase II study involving patients with unresectable, KIT-positive GIST that had progressed on treatment with imatinib and sunitinib, the median PFS and OS times were 5.2 and 11.6 months, respectively, while the one- and two-year survival rates were 50 and 29%, respectively (27). In a retrospective analysis of 32 patients, sorafenib exhibited significant clinical activity in a heavily pretreated group of patients with metastatic GIST resistant to imatinib, sunitinib and nilotinib (28). In a more recent study, patients with metastatic GISTs refractory to first-line imatinib and second-line sunitinib were treated at the discretion of their physician. The authors concluded that sorafenib had significant clinical activity in imatinib-resistant and sunitinib-resistant GISTs (34). In the present study, as in these studies, in heavily pretreated patients, the median PFS and OS times of the patients that received sorafenib were 7.2 and 15.2 months, respectively. Thus, the present study demonstrated the improved effect of sorafenib in patients with metastatic GIST who experience previous treatment failure.

In the present study, the duration of imatinib usage significantly affected PFS and OS. Demetri *et al* detected no association between the duration of imatinib treatment and survival (7). In the present study, the majority of the patients treated with sunitinib used imatinib for >12 months. Of these patients, those who received imatinib for longer had improved OS and PFS times than those who received it for a shorter time. There were no significant associations between the dose of imatinib and OS and PFS (P>0.05).

The current risk-group stratification according to Fletcher *et al* does not include the possible effect of the tumor site (32). Another recently suggested GIST risk-group stratification system takes the tumor site into account, as well as tumor size and the mitotic rate, dividing GISTs into possibly benign, uncertain or low malignancy potential and possibly malignant (35). In the present study, the majority of patients were classified as high-risk (76.0%) according to the NIH risk classification. This result was significantly higher than that reported by previous studies (36,37). There were no associations between tumor size, mitosis, risk category and PFS and OS. These differences may be explained by the progressive nature of the GISTs in patients receiving sorafenib treatment and should be evaluated with further studies.

In the present study, the clinical benefit rate of sorafenib treatment was 40%. This clinical benefit rate was lower than that in the phase II study reported by Kindler *et al* (27). Reichardt *et al* reported that 19% of patients who received sorafenib after the failure of imatinib, sunitinib and nilotinib in fourth-line treatment achieved partial remission, while 44% achieved disease stabilization (28). Based on the response rates achieved in the present study and these previous studies, sorafenib treatment may be accepted as clinically beneficial, although the limited experience with regard to response rates while using sorafenib treatment should be further evaluated with larger prospective series. Additionally, to determine the best practice in the third-line or fourth-line treatment, randomized, prospective comparative studies between sorafenib and other agents such as regorafenib should be conducted.

Italiano *et al* observed that albumin levels and KIT/PDGFRA mutational status were significantly associated with PFS, whereas performance status and albumin level were associated with OS (34). Furthermore, Heinrich *et al* indicated that sorafenib was more effective than imatinib or sunitinib for inhibiting the kinase activity of drug-resistant KIT mutants (26). As a limitation to the present study we were unable to determine the kinase mutations in our patients. In Turkey, it is not a routine practice to determine kinase mutations. Kinase mutations may explain the differences in the longevity of the PFS and OS and lower response rates in the present study. Therefore, an analysis of these mutations should be performed in further studies.

Sorafenib treatment is associated with several adverse effects. Fatigue, skin rashes and hematological toxicity were the most common side-effects in the present study. These side-effects are generally mild and may be managed by dose modulation. The toxicity profile reported in the present study was similar to that observed in previous studies, with the exception of hypertension (27,38). No serious treatment-related hypertension was observed with sorafenib and there was no treatment discontinuation.

In summary, sorafenib is an active and effective agent with a reasonable side-effect profile in the treatment of patients with gastrointestinal stromal tumors in third- or fourth-line treatments that are refractory to previous therapies. A significant number of patients with advanced GIST benefitted from sorafenib, with OS times exceeding one year. It was observed that the duration of imatinib usage was a significant independent prognostic factor for PFS and OS. Future prospective studies of sorafenib in GIST should investigate these factors to clarify the correlations of this clinical benefit.

## Figures and Tables

**Table I.** Patient characteristics.

Characteristics	Value
Age, years	
Median (range)	54.0 (16–82)
Gender, n (%)	
Male	17 (68.0)
Female	8 (32.0)
Tumor site, n (%)	
Stomach	6 (24.0)
Small intestine	16 (64.0)
Colon	1 (4.0)
Other sites	2 (8.0)
Histopathology, n (%)	
Spindle cell type	9 (36.0)
Epitheloid	7 (28.0)
Mixed type	9 (36.0)
Cd117 positivity, n (%)	
Cd117^+^, Cd34^+^	17 (68.0)
Cd117^+^, Cd34^−^	8 (32.0)
Site of metastases, n (%)	
Liver	11 (64.7)
Peritoneum	3 (17.6)
Lung	1 (5.9)
Others	2 (11.8)
Duration of imatinib usage, n (%)	
<6 months	4 (16.0)
>6 months	21 (84.0)

**Table II.** Tumor characteristics of 25 patients with gastrointestinal stromal tumors receiving sorafenib treatment.

Variables	Number of patients (%)
Tumor size, cm	
2–5	3 (12.0)
5–10	4 (16.0)
>10	18 (72.0)
Mitosis, HPF	
≤5/50	8 (32.0)
>6–10/50	5 (20.0)
>10/50	12 (48.0)
Fletcher risk categories	
Very low	0 (0.0)
Low	0 (0.0)
Intermediate	6 (24.0)
High	19 (76.0)

HPF, high power field.

**Table III.** Univariate analysis between clinopathological characteristics of the patient group and OS and PFS

Variables	PFS			OS		
	Median (months)	95% CI (months)	P-value	Median (months)	95% CI (months)	P-value
Age, years			0.052			0.482
<50	15.08	6.89–29.47		15.06	10.13–20.02	
>50	6.94	2.43–8.00		15.32	7.84–22.31	
Gender			0.866			0.858
Men	5.29	2.31–8.26		16.59	10.37–22.87	
Women	15.08	8.50–17.67		12.89	7.46–18.92	
Tumor site			0.008			0.223
Stomach	15.08	9.56–17.32		17.18	8.83–25.52	
Small intestine	6.29	5.08–6.49		15.12	12.63–21.60	
Colon	2.73	2.43–2.83		10.15	10.15–10.15	
Other sites	2.62	NA		9.86	NA	
Histopathology			0.499			0.179
Spindle cell type	3.02	2.53–6.14		17.77	NA	
Epitheloid	4.76	NA		11.99	NA	
Mixed type	15.08	8.14–30.35		19.37	15.93–22.80	
Tumor size, cm			0.709			0.811
2–5	4.76	NA		11.99	NA	
5–10	5.22	2.66–8.25		10.58	3.94–18.81	
>10	7.65	1.24–14.06		15.02	11.33–21.12	
Mitotic count, HPF			0.018			0.233
≤5/50	15.08	12.73–22.79		15.00	13.46–22.51	
>6–10/50	4.76	2.44–7.17		11.99	7.19–14.08	
>10/50	2.43	1.05–6.66		6.59	3.44–11.16	
Duration of imatinib usage, months			0.018			0.003
<6	3.10	1.15–5.64		4.20	2.78–8.56	
>6	7.60	3.85–11.34		17.60	12.78–23.68	
Response to sorafenib			0.000			0.007
PR-SD	12.08	11.36–20.76		15.87	11.10–34.51	
PD	5.60	4.12–6.83		10.56	3.80–20.17	

PFS, progression-free survival; OS, overall survival; PR, partial response; SD, stable disease; PD, progressive disease; NA, not available; HPF, high power field.

**Table IV.** The multivariate analysis between clinopathological characteristics of the patient group and OS and PFS.

Variables	PFS			OS		
	Hazard ratio	95% CI	P-value	Hazard ratio	95% CI	P-value
Duration of imatinib usage	0.079	0.007–0.895	0.04	0.058	0.005–0.65	0.021

PFS, progression-free survival; OS, overall survival.
